# Repeat procedures after pulsed field ablation for atrial fibrillation: *MANIFEST-REDO* study

**DOI:** 10.1093/europace/euaf012

**Published:** 2025-01-17

**Authors:** Daniel Scherr, Mohit K Turagam, Philippe Maury, Yuri Blaauw, Pepijn van der Voort, Petr Neuzil, Tobias Reichlin, Andreas Metzner, Johan Vijgen, Josef Kautzner, Serge Boveda, Ante Anic, Jim Hansen, Martin Manninger, Philipp Sommer, Frederic Anselme, Stephan Willems, Thomas Deneke, Roland Tilz, Daniel Steven, Reza Wakili, Pierre Jais, Moritoshi Funasako, Thomas Arentz, Anne Rollin, Bart A Mulder, Alexandre Ouss, Jan Petru, Thomas Kueffer, Marc D Lemoine, Pieter Koopman, Petr Peichl, Raquel Adelino, Zrinka Jurisic, Martin Ruwald, Anna-Sophie Eberl, Christian Sohns, Arnaud Savoure, Karin Nentwich, Melanie Gunawardene, Christian-Hendrik Heeger, Arian Sultan, Jan-Eric Bohnen, Jana Kupusovic, Nicolas Derval, Heiko Lehrmann, Emmanuel Ekanem, Vivek Y Reddy

**Affiliations:** Division of Cardiology, Department of Internal Medicine, Medical University of Graz, Auenbruggerplatz 15, 8036 Graz, Austria; Icahn School of Medicine at Mount Sinai, New York, NY, USA; Department of Cardiology, University Hospital Rangueil, Toulouse, France; I2MC, INSERM UMR 1297, Toulouse, France; Department of Cardiology, University of Groningen, University Medical Center Groningen, Groningen, The Netherlands; Catharina Hospital, Eindhoven, The Netherlands; Cardiology Department, Na Homolce Hospital, Homolka Hospital, Prague, Czech Republic; Inselspital—Bern University Hospital, University of Bern, Bern, Switzerland; University Heart & Vascular Center, University Medical Center Hamburg-Eppendorf, Hamburg, Germany; Department of Cardiology, Jessa Hospitals, Hasselt, Belgium; IKEM-Institute for Clinical and Experimental Medicine, Prague, Czech Republic; Heart Rhythm Department, Clinique Pasteur, Toulouse, France; Department for Cardiovascular Diseases, University Hospital Center Split, Split, Croatia; Department of Cardiology, Herlev-Gentofte University Hospital, Hellerup, Denmark; Division of Cardiology, Department of Internal Medicine, Medical University of Graz, Auenbruggerplatz 15, 8036 Graz, Austria; Clinic for Electrophysiology, Herz- und Diabeteszentrum NRW, Ruhr-University Bochum, Bad Oeynhausen, Germany; Department of Cardiology, Rouen Hospital, Rouen, France; Heart Center Bad Neustadt, Rhoen-Clinic Campus Bad Neustadt, Bad Neustadt an der Saale, Germany; Asklepios Hospital St. Georg, Hamburg, Germany; German Center for Cardiovascular Research (DZHK), Partner Site Hamburg/Kiel/Lübeck, Lübeck, Germany; University Heart Center Lübeck, Department of Rhythmology, University Hospital Schleswig-Holstein, Germany; Department for Electrophysiology, Heart Center University Hospital of Cologne, Cologne, Germany; Department of Medicine and Cardiology, Goethe University, Frankfurt, Germany; German Center for Cardiovascular Research DZHK, Partner Site Rhine-Main, Germany; IHU LIRYC, CHU Bordeaux, University of Bordeaux, Bordeaux, France; Cardiology Department, Na Homolce Hospital, Homolka Hospital, Prague, Czech Republic; Neuron Medical, Brno, Czech Republic; Department of Cardiology and Angiology, Medical Center and Faculty of Medicine - University of Freiburg, Freiburg, Germany; Department of Cardiology, University Hospital Rangueil, Toulouse, France; Department of Cardiology, University of Groningen, University Medical Center Groningen, Groningen, The Netherlands; Catharina Hospital, Eindhoven, The Netherlands; Cardiology Department, Na Homolce Hospital, Homolka Hospital, Prague, Czech Republic; Inselspital—Bern University Hospital, University of Bern, Bern, Switzerland; University Heart & Vascular Center, University Medical Center Hamburg-Eppendorf, Hamburg, Germany; Department of Cardiology, Jessa Hospitals, Hasselt, Belgium; IKEM-Institute for Clinical and Experimental Medicine, Prague, Czech Republic; Heart Rhythm Department, Clinique Pasteur, Toulouse, France; Department for Cardiovascular Diseases, University Hospital Center Split, Split, Croatia; Department of Cardiology, Herlev-Gentofte University Hospital, Hellerup, Denmark; Division of Cardiology, Department of Internal Medicine, Medical University of Graz, Auenbruggerplatz 15, 8036 Graz, Austria; Clinic for Electrophysiology, Herz- und Diabeteszentrum NRW, Ruhr-University Bochum, Bad Oeynhausen, Germany; Department of Cardiology, Rouen Hospital, Rouen, France; Heart Center Bad Neustadt, Rhoen-Clinic Campus Bad Neustadt, Bad Neustadt an der Saale, Germany; Asklepios Hospital St. Georg, Hamburg, Germany; German Center for Cardiovascular Research (DZHK), Partner Site Hamburg/Kiel/Lübeck, Lübeck, Germany; University Heart Center Lübeck, Department of Rhythmology, University Hospital Schleswig-Holstein, Germany; Department of Rhythmology, Cardiology and Intensive Care Medicine, Asklepios Klinik Altona, Hamburg, Germany; Department for Electrophysiology, Heart Center University Hospital of Cologne, Cologne, Germany; Department of Cardiology and Vascular Medicine, West German Heart and Vascular Center Essen, University Duisburg-Essen, Duisburg, Germany; Department of Medicine and Cardiology, Goethe University, Frankfurt, Germany; German Center for Cardiovascular Research DZHK, Partner Site Rhine-Main, Germany; IHU LIRYC, CHU Bordeaux, University of Bordeaux, Bordeaux, France; Department of Cardiology and Angiology, Medical Center and Faculty of Medicine - University of Freiburg, Freiburg, Germany; Icahn School of Medicine at Mount Sinai, New York, NY, USA; Icahn School of Medicine at Mount Sinai, New York, NY, USA; Cardiology Department, Na Homolce Hospital, Homolka Hospital, Prague, Czech Republic

**Keywords:** Atrial fibrillation, Atrial tachycardia, Electroporation, Pulsed field ablation, Pulmonary vein isolation

## Abstract

**Aims:**

Initial clinical studies of pulsed field ablation (PFA) to treat atrial fibrillation (AF) indicated a >90% durability rate of pulmonary vein isolation (PVI). However, these studies were largely conducted in single centres and involved a limited number of operators. We aimed to describe the electrophysiological findings and outcomes in patients undergoing repeat ablation after an initial PF ablation for AF.

**Methods and results:**

In the MANIFEST-REDO study, we investigated patients who underwent repeat ablation due to clinical recurrence—AF or atrial tachycardia (AT)—following first-ever PVI with a pentaspline PFA catheter (Farawave, Boston Scientific Inc.). At 22 centres, 427 patients (age 64 ± 11 years; 37% female) were included. Of note, the recurrent arrhythmia leading to the repeat ablation was paroxysmal AF (51%), persistent AF (30%), or AT (19%). At the repeat procedure, the PV reconnection rates were 30% (left superior pulmonary vein), 28% (left inferior pulmonary vein), 33% (right superior pulmonary vein), and 32% (right inferior pulmonary vein). In 45% of patients, all PVs were durably isolated at the beginning of the repeat procedure, with the previous use of any imaging or mapping modality being univariately associated with durable PVI. After a post-redo follow-up period of 284 (90–366) days, the primary effectiveness endpoint (freedom from documented AF/AT lasting ≥30 s after 3-month blanking without class I/III antiarrhythmic drugs or symptoms) was achieved in 65% of patients, with significant differences between groups (PAF 65% vs. PersAF 56% vs. AT 76%; *P* = 0.04). Persistent AF as recurrent arrhythmia after the initial PFA ablation predicted AT/AF recurrence after repeat ablation [hazard ratio 1.241 (95% confidence interval 1.534–1.005); *P* = 0.045]. The procedural complication rate was 2.8%.

**Conclusion:**

In repeat procedures for AF/AT performed after an index procedure with PFA for AF, PV reconnections are not uncommon. Repeat procedures can be performed safely and with an acceptable subsequent success rate.

## Graphical Abstract

**Graphical Abstract:**
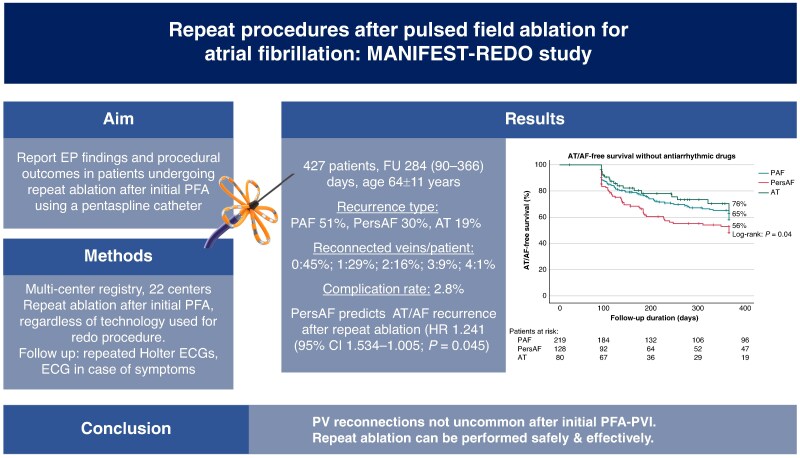

What’s new? The present study MANIFEST-REDO is among the first to evaluate procedural findings and clinical outcome in patients undergoing repeat ablation after AF/AT recurrence after initial AF ablation with the pentaspline PFA catheter. It has several important findings:

- Lesion durability in repeat ablation patients after PFA is limited in a real-world setting and may contribute to AF/AT recurrence, with 45% of patients having durable PVI at the repeat procedure start, and the previous use of any imaging modality being associated with durable PVI in this cohort.
- Repeat ablations for AF/AT after an initial PFA ablation can be performed with acceptable safety and efficacy, with a 65% clinical success rate in the first year and a 2.8% procedural complication rate.
- Persistent AF as recurrent arrhythmia after the initial PFA ablation is a predictor of worse clinical outcome after a repeat ablation.

## Introduction

Atrial fibrillation (AF) is the most prevalent sustained cardiac arrhythmia worldwide, significantly contributing to morbidity, mortality, and rising healthcare costs.^1^ Catheter ablation has emerged as an important therapy for patients with symptomatic AF.^1,2^ Pulmonary vein isolation (PVI) serves as the procedural standard. However, the durability of PVI after the initial ablation procedure remains a key limitation, necessitating repeat procedures in a significant subset of patients due to AF recurrence. This limitation underscores the need for advancements in catheter design and ablation techniques, not only for PVI but also for targeting extra-PV substrates of AF.^1–3^

Electroporation, also known as pulsed field ablation (PFA), is a new non-thermal ablation modality that employs high-voltage electrical pulses to selectively disrupt myocardial cell membranes.^3^ As opposed to thermal techniques, PFA minimizes the risk of collateral injury to adjacent anatomical structures, such as the oesophagus or the phrenic nerve, while still achieving effective lesion formation.^3–5^ Recently, innovative catheter designs, such as the pentaspline catheter, a multielectrode catheter optimized for PFA, have demonstrated significant safety, efficiency, and efficacy in achieving PVI during AF ablation procedures.^3–11^

The clinical utility of PFA has been demonstrated in multicentre studies. The MANIFEST-PF registry provides comprehensive data on the performance of PFA, highlighting its high procedural success rates and favourable safety profile.^12,13^ Similarly, findings from the EU-PORIA registry emphasize the applicability of PFA in diverse patient populations, demonstrating its effectiveness in real-world settings.^14^ Long-term studies have reinforced the durability of PFA-induced PVI, showing reduced AF recurrence rates over extended follow-up periods. Importantly, the capability to reapply PFA energy during repeat ablation procedures without compromising safety further underscores its versatility and clinical potential.^10,11^ However, there is limited data on procedural findings and especially on outcomes in patients undergoing re-ablation for AF or atrial tachycardia (AT) recurrence after an initial PF ablation for AF.^15–20^

This multicentre study (MANIFEST-REDO) aims to evaluate the outcomes of catheter re-ablation in patients with AF/AT who previously underwent PF AF ablation using the pentaspline catheter. Specifically, procedural success, safety, and lesion durability findings during re-ablation, as well as factors contributing to AF/AT recurrence, are investigated.

## Methods

### Study design

*MANIFEST-PF* is an international, prospective, patient-level registry involving 24 European centres that initiated the post-approval clinical application of the pentaspline PFA catheter (Farawave, Boston Scientific Inc.) for AF ablation.^12,13,21,22^ Patients (age ≥ 18 years) who underwent first-ever PFA for AF between October 2021 and January 2024 were included. Of note, most operators had no clinical experience with performing PFA prior to these patients, so the learning curve was incorporated into this registry. The *MANIFEST-PF* registry adhered to the principles of the Declaration of Helsinki, and the waiver of consent was approved by the Ethics Committee at Homolka Hospital.

### Initial ablation procedure

The procedure methodology and post-procedural monitoring of patients included in the *MANIFEST-PF* registry have been detailed in previous publications.^12,13,21,22^ In brief, the PFA procedure was conducted under deep sedation (without endotracheal intubation) or general anaesthesia with intubation. Procedures were typically performed with uninterrupted oral anticoagulation and systemic heparinization before transseptal puncture. Electroanatomic mapping and intracardiac echocardiography (ICE) imaging were used at operator discretion. Following transseptal puncture, the deflectable PFA sheath (Faradrive, Boston Scientific) was advanced into the left atrium (LA) using a 0.035 in super-stiff guidewire, and baseline electrical potentials were recorded from all pulmonary veins (PVs) using the pentaspline PFA catheter. Pulmonary vein isolation was performed with 2 applications per PV in both basket and flower configurations, and then the basket/flower was rotated ≈36° to change the spline orientation and another two applications were delivered (total of 8 per PV). Additional PFA applications were administered as deemed necessary by the operator. Confirmation of PVI typically relied on electrograms recorded from the pentaspline PFA catheter. Adjunctive ablation of the posterior wall, roof, mitral isthmus, cavo-tricuspid isthmus, and other sites was typically performed with PFA, though a commercially available radiofrequency (RF) ablation catheter may have been used per operator discretion. The use of post-procedure antiarrhythmic drugs (AADs) was at the discretion of the treating physician for a short duration, and oral anticoagulation was continued in accordance with AF guidelines.^1,2^

Patients commonly attended follow-up visits at 3, 6, and 12 months post-procedure, during which evaluations were conducted to assess adverse events, AF-related symptoms, and recurrence of atrial arrhythmias with either 12-lead electrocardiogram, 24-h Holter monitoring, or cardiac implantable electronic device interrogation, as determined by the physician’s discretion.

### Repeat ablation procedures

If patients had a symptomatic AF/AT recurrence >3 months after the initial AF ablation procedure, they were deemed eligible for a repeat ablation procedure. With regard to sedation, anticoagulation, imaging, and mapping, repeat ablation procedures were performed under the same circumstances as the initial procedures.

For the repeat procedure, the ablation system was chosen at the operators’ discretion. As for the ablation itself during the repeat procedure, the following energy forms were used in this study: RF ablation, cryoballoon ablation, PFA with the pentaspline catheter, focal PFA, and/or alcohol ablation of the vein of Marshall.

After transseptal puncture, confirmation of PVI typically relied on electrograms recorded from either the pentaspline PFA catheter or from a multipolar mapping catheter. If PVI or any lesion targeted during the initial procedure was incomplete, ablation of the PVs or the respective substrate was performed with the designated ablation system. Adjunctive ablations of the posterior wall, roof, mitral isthmus, cavo-tricuspid isthmus, and other LA sites were performed per operator discretion. The use of post-procedure AADs was at the discretion of the treating physician for a short duration and oral anticoagulation was continued in accordance with AF guidelines.^1,2^ Patients’ follow-up was conducted as described above.^12,13,21,22^

### Study endpoints

The primary effectiveness endpoint was freedom from documented AF/AT lasting ≥30 s (after 3-month blanking), without class I/III AADs or symptoms. The secondary effectiveness endpoint was freedom from AF/AT lasting ≥30 s (after 3-month blanking) with or without the necessity for class I/III AADs.

The primary safety outcome encompassed a composite of acute events (occurring within 7 days post-procedure) and chronic major adverse events (occurring >7 days post-procedure).^12,13,21,22^

### Statistical analysis

Continuous variables were expressed as mean ± SD or median (interquartile range) and compared nonparametric Kruskal–Wallis tests. All comparisons among groups were performed using the Student’s *t*-test if the data were normally distributed or the Wilcoxon rank-sum test if the data were not normally distributed. Categorical variables were presented as counts/percentages and compared using *χ*^2^ test or Fisher’s exact test (expected cell count <5).

Kaplan–Meier survival curves were utilized for primary and secondary effectiveness outcomes, with treatment groups compared using the log-rank test. Cox proportional hazards modelling was conducted to generate hazard ratios (HRs) and corresponding 95% confidence intervals (CIs) for time-to-event analyses. Covariates included in the adjusted model were selected based on a clinically plausible association with AT/AF recurrence and PVI durability, and if a univariate association of *P* < 0.1 was present. Tested variables were time to repeat ablation, PVI durability, gender, body mass index (BMI) > 35, posterior wall isolation durability, persistent AF as initial arrhythmia, coronary artery disease, diabetes mellitus, hypertension, heart failure, LA diameter, left ventricular ejection fraction (LVEF), CHA_2_DS_2_-VASc score, size of pentaspline catheter, persistent AF as arrhythmia at repeat ablation, use of any imaging, or electroanatomic mapping modality used at initial procedure. All tests were two tailed, with *P* < 0.05 considered statistically significant. SPSS software (version 29.0, IBM Corp.) was employed for all analyses.

## Results

### Patient characteristics

At 22 centres, 427 patients (age 64 ± 11 years; 37% female) who were scheduled for repeat ablation because of AF/AT recurrence after a previous PF ablation for AF with the pentaspline catheter were included. *Table 1* shows the baseline characteristics of the patients grouped by their recurrent arrhythmia after their initial ablation that led to the repeat ablation. Of note, the recurrent arrhythmia was paroxysmal AF (PAF; 219 patients, 51%), persistent AF (PersAF; 128 patients, 30%), or AT (80 patients; 19%).

**Table 1:** Baseline characteristics of repeat ablation patients grouped by atrial tachyarrhythmia leading to the repeat ablation

	Paroxysmal AF (*n* = 219)	Persistent AF (*n* = 128)	Atrial tachycardia (*n* = 80)	*P* value
Age (years)	63 ± 11	63 ± 12	66 ± 11	0.080
Female gender [*n* (%)]	79 (36)	47 (37)	30 (38)	0.663
BMI (kg/m^2^)	28 ± 5	28 ± 5	28 ± 5	0.524
CHA_2_DS_2_-VASc score	2.0 ± 1.4	2.2 ± 1.5	2.6 ± 1.5	0.014
CHF [*n* (%)]	17 (8)	19 (15)	18 (23)	0.002
Hypertension [*n* (%)]	109 (50)	73 (57)	52 (65)	0.051
Diabetes mellitus [*n* (%)]	32 (15)	18 (14)	17 (21)	0.320
Prior stroke/TIA [*n* (%)]	24 (11)	9 (7)	2 (3)	0.041
Sleep apnoea [*n* (%)]	14 (6)	11 (9)	7 (9)	0.674
Chronic obstructive pulmonary disease [*n* (%)]	11 (5)	6 (5)	6 (8)	0.644
CAD [*n* (%)]	26 (12)	21 (16)	16 (20)	0.170
LVEF (%)	58 ± 8	55 ± 11	57 ± 9	0.037
LA diameter (mm)	43 ± 7	46 ± 7	46 ± 7	0.009
Previous history of CTI-dependent flutter [*n* (%)]	24 (11)	26 (20)	20 (25)	0.005
Left common PV ostium [*n* (%)]	24 (11)	14 (11)	7 (9)	0.839
Paroxysmal AF at initial procedure [*n* (%)]	119 (54)	46 (36)	36 (45)	<0.001
Class I/III AAD [*n* (%)]	76 (35)	34 (27)	38 (48)	0.023
Oral anticoagulation [*n* (%)]	212 (97)	122 (95)	77 (96)	0.451
Pentaspline catheter size at first procedure:				0.285
31 mm [*n* (%)]	161 (74)	101 (79)	56 (70)	
35 mm [*n* (%)]	58 (26)	27 (21)	24 (30)	
PVI at first procedure [*n* (%)]	219 (100)	128 (100)	80 (100)	1.00
Posterior wall ablation at first procedure [*n* (%)]	54 (25)	52 (41)	34 (43)	<0.001
Other ablation at first procedure [*n* (%)]	28 (13)	22 (17)	26 (33)	<0.001

AAD, antiarrhythmic drug; BMI, body mass index; CAD, coronary artery disease; CHF, congestive heart failure; CTI, cavo-tricuspid isthmus; LA, left atrium; LVEF, left ventricular ejection fraction; PVI, pulmonary vein isolation; TIA, transient ischaemic attack.

There was a difference between groups with regard to the CHA_2_DS_2_-VASc score (PAF 2.0 ± 1.4 vs. PersAF 2.2 ± 1.5 vs. AT 2.6 ± 1.5; *P* = 0.014) and LA diameter (PAF 43 ± 7 mm vs. PersAF 46 ± 7 mm vs. AT 46 ± 7 mm; *P* = 0.009). The proportion of patients who had undergone the initial ablation with the 35 mm pentaspline catheter was not different between groups. However, there was a significant difference in patients who had undergone posterior wall isolation and/or additional LA substrate ablation during the first procedure, with the proportion of patients being the highest in the AT group (*Table 1*). Furthermore, there was a difference in class I/III AAD use at the time of repeat ablation (PAF 35% vs. PersAF 27% vs. AT 48%; *P* = 0.023).

### Procedural characteristics and safety outcomes of repeat ablation

Patients underwent repeat ablation 279 ± 171 days after the initial PFA procedure. The procedural characteristics for the repeat ablation based on the recurrent arrhythmia after the initial procedure are shown in *Table 2*. Of note, in patients with AT, ICE imaging and electroanatomic mapping wer used in a higher proportion of patients than in patients presenting with PAF or PersAF. Furthermore, extra-PV targets were ablated in a higher proportion of AT patients, leading to longer procedure and fluoroscopy duration. However, there was no difference between groups with regard to the energy form used (*Table 2*). The procedural complication rate was 2.8%: vascular complications (*n* = 5), pericardial effusion (*n* = 4), atrio-ventricular block (*n* = 1), stroke (*n* = 1), and LAA isolation (*n* = 1).

**Table 2:** Procedural characteristics of repeat ablation patients

	Paroxysmal AF (*n* = 219)	Persistent AF (*n* = 128)	Atrial tachycardia/flutter (*n* = 80)	*P* value
General anaesthesia [*n* (%)]	25 (11)	28 (22)	20 (25)	0.003
ICE Imaging [*n* (%)]	78 (36)	26 (20)	36 (45)	<0.001
Electroanatomic mapping [*n* (%)]	169 (77)	101 (79)	73 (90)	<0.001
Energy form [*n* (%)]				0.714
Pentaspline PFA	72 (33)	40 (31)	27 (34)	
RF	122 (56)	71 (55)	45 (56)	
Cryo	4 (2)	0 (0)	0 (0)	
Focal PFA	16 (7)	10 (8)	5 (6)	
Vein of Marshall	5 (2)	7 (5)	3 (4)	
Durable PVI at procedure start [*n* (%)]	91 (42)	56 (44)	44 (55)	0.385
Ablation targets [*n* (%)]				
PVI	142 (65)	75 (59)	47 (59)	0.118
Posterior wall	65 (30)	66 (52)	32 (40)	<0.001
Other LA substrate	114 (52)	69 (54)	63 (79)	0.012
Fluoroscopy time (min)	12 ± 9	15 ± 7	20 ± 12	<0.001
Procedure time (min)	91 ± 27	103 ± 37	107 ± 40	<0.001
Procedural complications [*n* (%)]	6 (3)	2 (2)	4 (5)	0.11

ICE, intracardiac echocardiography; LA, left atrium; PFA, pulsed field ablation; PVI, pulmonary vein isolation; RF, radiofrequency.

### Pulmonary vein isolation and posterior wall isolation durability

**Figure 1:**
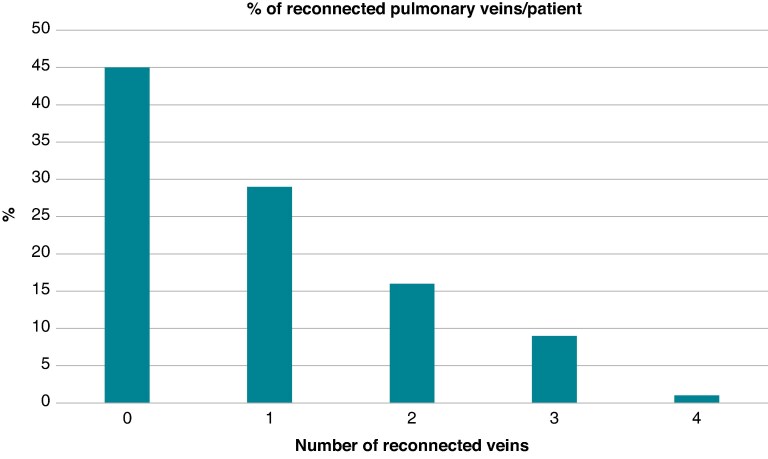
Percentage (respective number: *x*-axis) of reconnected veins (respective number: *y*-axis) per patient during repeat ablation.

At the repeat procedure, the PV reconnection rates were 29% (left superior pulmonary vein, LSPV), 27% (left inferior pulmonary vein, LIPV), 32% (right superior pulmonary vein, RSPV), and 31% (right inferior pulmonary vein, RIPV). In 45% of patients, all PVs were durably isolated at the beginning of the repeat procedure (PAF 42%, PersAF 44%, AT 55%; *P* = ns). Patients had 0 (45%), 1 (29%), 2 (16%), 3 (9%), or 4 (1%) reconnected veins (*Figure 1*). Notably, of all univariate associations tested, there was no association between time from initial to repeat ablation and the freedom from PV reconnection (time from initial to repeat ablation and durable PVI rates: <90 days: 35%; 90 days to 6 months: 49%; 6–12 months: 39%; >12 months: 39%; *P* = 0.454). However, the use of any form of (pre)procedural imaging (ICE, CT, electroanatomic mapping, rotational angiography) to guide the initial ablation was associated with a higher PVI durability rate (47% vs. 37%; *P* = 0.036).

Of the 140 patients who underwent posterior wall ablation during the initial ablation procedure and underwent repeat procedure, 64 (46%) presented with complete posterior wall isolation, with a higher persistent isolation rate in patients who had undergone any form of (pre)procedural imaging to guide the initial ablation (50% vs. 42; *P* = 0.05).

### Effectiveness outcome

**Figure 2:**
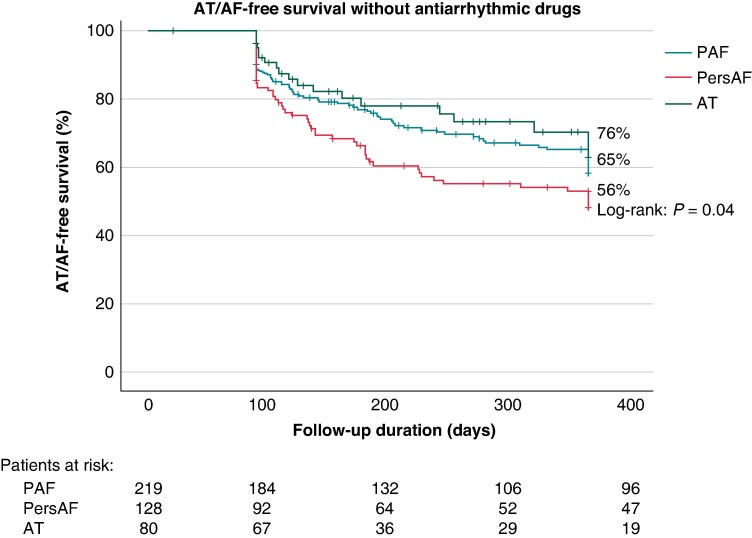
Kaplan–Meier estimate of primary effectiveness endpoint of freedom from documented AF/AT lasting ≥30 s (after 3-month blanking), without class I/III antiarrhythmic drugs or symptoms. AT, atrial tachycardia; PAF, paroxysmal AF; PersAF, persistent AF.

**Figure 3:**
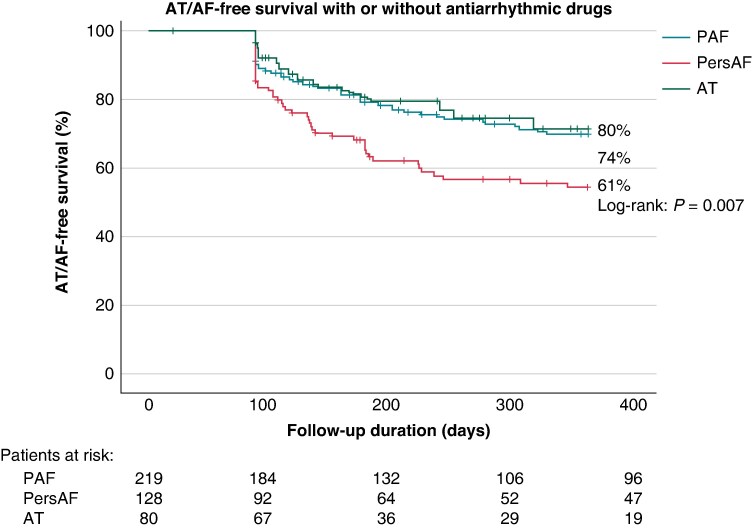
Kaplan–Meier estimate of secondary effectiveness endpoint of freedom from documented AF/AT lasting ≥30 s (after 3-month blanking), with or without class I/III antiarrhythmic drugs or symptoms. AT, atrial tachycardia; PAF, paroxysmal AF; PersAF, persistent AF.

After a follow-up period of 284 (90–366) days, the primary effectiveness endpoint (freedom from documented AF/AT lasting ≥30 s after 3-month blanking without class I/III AADs or symptoms) was achieved in 65% of patients, with significant differences between groups (PAF 65% vs. PersAF 56% vs. AT 76%; *P* = 0.04) (*Figure 2*). There was no association between time from initial to repeat ablation and the primary effectiveness endpoint (time from initial to repeat ablation and success rates: <90 days: 65%; 90 days to 6 months: 61%; 6–12 months: 61%; >12 months: 70%; *P* = ns). The secondary effectiveness endpoint (freedom from documented AF/AT lasting ≥30 s after 3-month blanking with or without class I/III AADs or symptoms) was achieved in 71% (PAF 74% vs. PersAF 61% vs. AT 80%; *P* = 0.007) (*Figure 3*).

In the subgroup of patients with persistent AF, there was borderline significance in secondary effectiveness with the use of posterior wall ablation (68% vs. 51%, *P* = 0.07). In multivariate analysis [including the univariately associated diabetes mellitus (*P* = 0.039), persistent AF as initial arrhythmia (*P* = 0.04), and persistent AF as recurrent arrhythmia (*P* = 0.067)], persistent AF as recurrent arrhythmia after the initial PFA ablation predicted AT/AF recurrence after repeat ablation [HR 1.241 (95% CI 1.534–1.005; *P* = 0.045)] (*Table 3*).

**Table 3:** Multivariate predictors of AF/AT recurrence after repeat ablation

	HR	95% CI	*P* value
Persistent AF as recurrent arrhythmia after the initial PFA ablation	1.241	1.534–1.005	0.045
Persistent AF as arrhythmia before the initial PFA ablation	1.000	1.004–0.996	0.863
Absence of diabetes mellitus	0.745	1.106–0.502	0.144

AF, atrial fibrillation; AT, atrial tachycardia; CI, confidence interval; HR, hazard ratio.

## Discussion

The present study MANIFEST-REDO is among the first to evaluate procedural findings and clinical outcome in patients undergoing repeat ablation after AF/AT recurrence after an initial PFA AF ablation with the pentaspline catheter. It has several important findings: (i) lesion durability in patients undergoing repeat ablation after initial PFA procedure is limited in a real-world setting and may contribute to AF/AT recurrence; (ii) repeat ablations for AF/AT after an initial PFA ablation can be performed with acceptable safety and efficacy; and (iii) persistent AF as recurrent arrhythmia is a predictor of worse clinical outcome after a repeat ablation.

### Lesion durability and pulmonary vein reconnection rates

The durability of PVI is a critical determinant of successful AF ablation. Various studies have investigated PVI durability after PFA ablation with the pentaspline catheter and showed excellent results.^23,24^ However, there is limited data on PVI durability in repeat ablation patients, bearing in mind that most studies, including ours, present PVI and posterior wall isolation durability data only in patients with AF/AT recurrences, therefore underestimating the true durability rates. The early experience from Tohoku *et al.*^16^ pointed to a PV reconnection rate of 9% in a limited patient cohort. On a per patient basis, persistent durable isolation of all four PVs was recorded in 19 (76%) patients. These single-centre findings, which in terms of PVI durability differ to the findings in our study as well as to the findings by Lemoine *et al.*,^19^ may be due to the limited patient number and the concentration in operator expertise possible in single-centre studies, as reported by Tohoku *et al.* In contrast, Lemoine *et al.* reported that of 82 initially isolated PVs after PFA–PVI, 31 (38%) were reconducting during repeat ablation and 73% of investigated patients showed at least one reconnected PV.^19^ Similarly, Maurhofer *et al.*^20^ found that in patients undergoing repeat ablation with PFA, 60% of patients had at least one reconnected PV and Kueffer *et al.*^15^ reported 62% in a sub-analysis of the EU-PORIA registry. Prior operator experience with cryoballoon ablation was associated with a higher PVI durability compared to operators with only point-by-point RF experience (76% vs. 60%; *P* < 0.001). Neither the operators’ cumulative experience in AF ablation nor the size of the PFA device used (31 mm vs. 35 mm) had an impact on subsequent lesion durability. While the PVI durability rate in PF ablation has been reported to be excellent, especially with the pentaspline catheter, and even in repeat ablation patients higher in post PFA patients than in post-cryoablation patients or post RF patients,^9^ future developments in PFA technology should focus on improving PVI durability. Our limited data showed that in patients undergoing repeat ablation, the use of any form of imaging or mapping was associated with an increased PVI durability rate. As previously suggested, the use of ICE may be helpful for PVI, but the absence of randomization is an important confounder that limits confidence in this observation.^25^ However, our study only investigated patients with clinical recurrence. Whether full integration of PFA technologies in 3D electroanatomic mapping systems or use of ICE will really improve PVI durability and therefore potentially clinical outcomes remains to be investigated. Badertscher *et al.*^26^ published their early experience on the impact of non-fully 3D integrated PF ablation and found that the use of a pentaspline PFA system with no mapping was associated with a significant decrease in procedural characteristics, while AF recurrence was not significantly different even if mapping was used. The routine use of mapping for PFA–PVI may be helpful. However, this needs to be investigated with fully integrated PF catheter systems that allow for contact assessment and lesion tracking.

### Procedural efficacy and safety

The MANIFEST-PF registry provides robust evidence on procedural outcomes for PFA, including data on repeat ablations.^12,13,21,22^ This registry underscored the consistent safety profile of the pentaspline catheter, reporting a complication rate of <3% even in patients undergoing re-ablation. The electroporation mechanism of PFA, which induces disruption of cardiomyocytes without thermal injury, emerges as a key factor in minimizing risks such as oesophageal or phrenic nerve damage.^27^ Several trials have validated the clinical efficacy of PFA ablation, which was shown to be at least equivalent to other energy modalities.^27^

Furthermore, our study highlights that repeat ablation for AF/AT after an initial PF ablation leads to acceptable success rates, especially in patients with PAF or AT as a recurrent arrhythmia.

Despite promising results, several challenges persist in the context of repeat ablations following PFA. Variability in lesion durability remains a concern, influenced by patient-specific factors such as anatomical variability and comorbid conditions. While our study showed a near-significant effectiveness of posterior wall ablation in PersAF patients, this finding is at best hypothesis generating: this near-significant finding was achieved in patients undergoing repeat (not initial) ablation, with difference in subgroups in terms of baseline characteristics, lesion set during initial ablation, and ablation energy used during repeat ablation.

### Limitations

This multicentre cohort study, involving 427 patients undergoing redo ablation of AF following a prior PFA ablation, has several limitations. Firstly, the absence of a control group restricts the ability to draw comparisons with other ablation techniques. The true PVI durability rate in the overall cohort of patients undergoing PFA cannot be concluded. Furthermore, the study does not provide direct evidence regarding the relative efficacy or safety of PFA in the redo ablation setting.

Secondly, the non-continuous nature of the follow-up could lead to an underestimation of arrhythmia recurrence, particularly in asymptomatic patients or those experiencing late PV reconnections. Continuous monitoring would provide a more accurate depiction of long-term outcomes.

Additionally, the multicentre design introduces heterogeneity in procedural techniques and operator experience across participating centres. This variability could influence the study results and limit their reproducibility. Future research with standardized protocols and continuous follow-up is essential to address all these limitations.

## Conclusions

In repeat procedures for AF/AT performed after an index procedure with PFA for AF, PV reconnections are not uncommon. Repeat procedures can be performed safely and with an acceptable subsequent success rate. Persistent AF as the recurrent arrhythmia is a predictor of a lower success rate during follow-up.
